# Dietary Reversal Ameliorates Short- and Long-Term Memory Deficits Induced by High-fat Diet Early in Life

**DOI:** 10.1371/journal.pone.0163883

**Published:** 2016-09-27

**Authors:** Catrina Sims-Robinson, Anna Bakeman, Elizabeth Bruno, Samuel Jackson, Rebecca Glasser, Geoffrey G. Murphy, Eva L. Feldman

**Affiliations:** 1 Department of Neurology, Medical University of South Carolina, Charleston, South Carolina, 29425, United States of America; 2 Department of Neurology, University of Michigan, Ann Arbor, Michigan, 48109, United States of America; 3 Department of Molecular and Integrative Physiology, University of Michigan, Ann Arbor, Michigan, 48109, United States of America; 4 Department of Molecular and Behavioral Neuroscience Institute, University of Michigan, Ann Arbor, Michigan, 48109, United States of America; Hospital Infantil Universitario Nino Jesus, SPAIN

## Abstract

A high-fat diet (HFD), one of the major factors contributing to metabolic syndrome, which is associated with an increased risk of neurodegenerative diseases, leads to insulin resistance and cognitive impairment. It is not known whether these alterations are improved with dietary intervention. To investigate the long-term impact of a HFD on hippocampal insulin signaling and memory, C57BL6 mice were placed into one of three groups based on the diet: a standard diet (control), a HFD, or a HFD for 16 weeks and then the standard diet for 8 weeks (HF_16_). HFD-induced impairments in glucose tolerance and hippocampal insulin signaling occurred concurrently with deficits in both short- and long-term memory. Furthermore, these conditions were improved with dietary intervention; however, the HFD-induced decrease in insulin receptor expression in the hippocampus was not altered with dietary intervention. Our results demonstrate that memory deficits due to the consumption of a HFD at an early age are reversible.

## Introduction

Nearly 35% of adults over the age of 20 and 50% of adults over the age of 60 have metabolic syndrome (MetS) [1]. Unfortunately, there is no consensus on diagnosis criteria for MetS in juveniles (under the age of 18); however, the rates correlate with the obesity epidemic [2]. MetS is the term designating the presence of multiple comorbidities, such as obesity, hyperinsulinemia, hyperglycemia, dyslipidemia, and hypertension. Poor dietary choices, such as the consumption of a high-fat (HF) diet (HFD), contribute heavily to many of these comorbidities [3]. Factors associated with MetS negatively impact cognition and are risk factors for dementia [4,5]. A HFD disrupts learning and memory performance in rodents [6–8]. Given that MetS is epidemic and tied to impaired cognition, understanding the long-term impact of a HFD on the hippocampus, a key structure involved in learning and memory, is of paramount importance.

It is likely that several pathways are involved in the mechanisms underlying diet-induced changes on cognition [9]. The insulin signaling pathway may play an important role in diet-induced hippocampal dysfunction. In the periphery, insulin binds to the insulin receptor (InsR), which leads to a conformational change and the activation of tyrosine kinases [10,11]. Tyrosine kinase activation leads to the rapid phosphorylation (p) of ‘docking proteins’ such as the InsR substrate 1 (IRS_1_) [12]. pIRS_1_ plays a critical regulatory role in insulin signaling and triggers multiple signaling pathways, such as the phosphatidylinositol 3-kinase (PI3K)/protein kinase B (AKT) pathway, to activate downstream insulin signaling [13].

A HFD is associated with peripheral insulin resistance, which occurs following hyperinsulinemia and InsR desensitization [14,15]. Animal models and humans with insulin resistance demonstrate a reduction in the IRS_1_/PI3K/AKT pathway [16]. This is mediated by the serine pIRS_1_, which minimizes insulin signaling by reducing the ability of IRS_1_ to attract PI3K and activate AKT (pAKT) [17,18].

The molecular mechanisms of insulin resistance in the CNS are not well characterized. Studies have focused on the IRS_1_/pAKT pathway, similar to peripheral insulin resistance [14,19]. In the CNS, insulin is involved in numerous functions, including neuronal survival, synaptic maintenance, dendritic arbor development, learning, and memory [20,21]. InsRs are localized in hippocampal neuron cell bodies and synapses, highlighting its role in cognition. Furthermore, therapeutic strategies to enhance insulin signaling in the CNS have been widely investigated over the past decade and demonstrate promising results for improving cognition [22,23].

Deficits in cognition in mice given a HFD early in life have been reported [24–26]. Given the importance of insulin signaling in cognition, it is imperative to understand the long-term impact of a HFD on insulin signaling in the hippocampus and the associated changes in memory. In this study, the reversibility/persistency of the effects of a HFD in C57BL6 (B6) mice was assessed. Hippocampal-dependent short- and long- term memory was evaluated using novel object recognition (NOR) [27] and Morris Water Maze (MWM) [28,29], respectively. After returning the mice to a normal diet, insulin sensitivity both in the periphery and hippocampus and the ability of dietary intervention to reverse memory deficits were evaluated.

## Materials and Methods

### Animals

Male B6 mice, purchased from Jackson Laboratory (Bar Harbor, Maine) at 4 weeks of age, were placed on either the standard diet consisting of 10% kcal from fat (Research Diets Inc.; #D12450B, New Brunswick, NJ) or a HFD with 54% kcal from fat (Research Diets Inc.; D05090701) ad libitum. A comparison of the caloric content as well as the food intake for one week after 24 weeks of diet are presented as supporting data (S1 Table). To evaluate the long-term consequences of a HFD, some HFD mice were switched to the standard diet after 16 weeks of the HFD. Thus, this study consisted of three groups: 1) standard diet (control; CTRL), 2) HFD, and 3) HFD for 16 weeks then standard diet for the remainder of the study (HF_16_).

Mice were housed in a pathogen-free environment. All protocols/procedures were approved by the University of Michigan Committee on the Use and Care of Animals or the Medical University of South Carolina Institutional Animal Care and Use Committee, and are in compliance with the university guidelines, state and federal regulations, and the standards of the “Guide for the Care and Use of Laboratory Animals.” Animal Welfare Assurance Numbers on file with the NIH Office of Laboratory Animal Welfare (OLAW) are A3114-01 (University of Michigan) and A3428-01 (Medical University of South Carolina). Both universities are accredited by the Association for the Assessment and Accreditation of Laboratory Animal Care International (AAALAC, Intl.).

### Metabolic Phenotyping

Body weights were monitored monthly using a standard laboratory scale. Impaired glucose tolerance testing was performed after 12 and 24 weeks of diet. Following a 4 hour fast, blood glucose levels were obtained using a standard glucometer (One Touch Ultra, Milpitas, CA) at 0, 15, 30, 60, and 120 minutes after the administration of a bolus dose of glucose (1g glucose/ kg body weight, intraperitoneal). Fasting plasma insulin levels were determined by the Mouse Metabolic Phenotyping Centers (MMPC; Vanderbilt University, Nashville, TN)

### Tissue Preparation

Mice were euthanized according to our previously published protocols [30] with a sodium pentobarbital overdose following a 4 hour fast. The hippocampus from each hemisphere from one cohort of mice were dissected and used fresh for *ex vivo* insulin stimulation as described below. The hippocampus from each hemisphere in a separate cohort of mice were dissected and flash frozen in liquid nitrogen and stored at -80°C until use for western immunoblotting.

### *Ex vivo* insulin stimulation

*Ex vivo* insulin stimulation was performed per our previously published protocol [30]. Briefly, the hippocampus from each hemisphere was minced and placed into separate microcentrifuge tubes in Neurobasal medium containing 5 mM glucose, 2.5 μg/ml albumin, 10 μg/ml apo-transferrin, 0.1 μg/ml biotin, 15 μg/ml D-galactose, 7 ng/ml progesterone, 16 μg/ml putrescine, 4 ng/ml selenium, 3 ng/ml β-estradiol, 4 ng/ml hydrocortisone, 3 μg/ml catalase, and 2.5 μg/ml superoxide dismutase. The tubes were placed at 37°C for 45 minutes to stabilize prior to stimulation with 20 nM insulin for 30 minutes. The hippocampus from one hemisphere was used as the control (no insulin) while the other hemisphere was stimulated with insulin. The media was removed following centrifugation at 1000 rpm and the tissue processed for western immunoblotting as described below.

### Western immunoblotting

Western immunoblotting was performed per our previously published protocol [30]. Briefly, the tissue lysates were separated by SDS-PAGE and transferred to a nitrocellulose membrane. TBS with Tween-20 supplemented with 5% BSA was used to block the membrane and to dilute the antibodies. Polyclonal antibodies against pAKT (serine 473), tyrosine (Y1222) pIRS_1_, serine (s) pIRS_1_ (307 and 636/639, IRS_1_, InsR (all from Cell Signaling Technology, Danvers, MA), and tubulin (Abcam, Cambridge, MA), as well as the appropriate horseradish peroxidase-conjugated secondary antibodies (Santa Cruz Biotechnology, Inc.), were used for western immunoblotting. The signal was visualized using LumiGLO enhanced chemiluminescence reagent (Cell Signaling Technology, Danvers, MA). Images were captured using the Chemidoc XRS system and analyzed by Quantity One software (Bio-Rad Laboratory, Hercules, CA).

### Novel Object Recognition Test

The NOR test was performed in a circular, opaque-colored open field (46 cm diameter x 38 cm height) as previously described [31]. A camera mounted above the open field and LimeLight 3 Video tracking software system (ActiMetrics, Wilmette, IL) were used to record the movements of the mouse throughout the test. On day one (habituation), the mice were allowed to explore the empty arena three times (5 minutes each). On day two (exploration phase), the mice were allowed to explore two identical objects located in opposite quadrants (5 minutes) before being returned to their home cage. Following a 30–45 minute delay, the mice were returned to the arena with one object identical to those used in the exploration phase and a second novel object located in opposite quadrants. The mice were allowed to explore the objects (5 minutes). The objects used for the NOR test were small Legos of similar sizes. The arena and objects were cleaned thoroughly with 70% ethanol after each trial. The time the mice spent exploring each object was recorded by a trained observer using the LimeLight program. Exploration of an object was recorded when one or more of the following instances occurred: the mouse’s head was oriented directly towards the object, the mouse was directly touching the object, or the mouse was sniffing the object. Object recognition memory was defined as the ratio of exploration time for the novel object (T_N_) over the exploration time for the novel plus familiar object (T_F_) (exploration ratio = T_N_ / (T_N_ +T_F_)).

To determine the reversibility of recognition memory deficits, a within-subjects comparison test was performed in a separate cohort of mice by conducting the NOR test 1 week after arrival from Jackson Laboratories and prior to any dietary change to generate a baseline for each mouse. Mice that did not spend significantly more than 50% of the time (chance level) exploring the novel object were excluded from the remainder of the study. Mice that spent significantly more than 50% of time exploring the novel object were randomly assigned by cage to either the CTRL or HF_16_ group. NOR was performed at 0 (before the start of diet), 12, and 22 weeks after the start of the diet.

### Morris Water Maze Test

The MWM test was performed as previously described [32]. Briefly, the water was temperature controlled at 25 (±2°C) and made opaque using non-toxic white paint to hide the Plexiglas escape platform (10 cm), which is submerged 0.5 cm below the water surface in the center of one of the quadrants in a 1.2 m diameter pool. High-contrast pictures were used as distal cues on the walls surrounding the pool. Indirect white lights were used to light the room (200 lux in center of pool). Ten days prior to MWM training, mice were handled for 2–3 minutes per day. Each training trial began with the mouse on the platform for 15 seconds. The mouse was placed into the water from a starting position pseudo-randomly chosen among 6 start positions facing the wall of the pool and allowed to search until finding the platform or until 60 seconds had elapsed. At the completion of each trial, the mouse was allowed to remain on the platform for 15 seconds. Mice were given 5 trials per day for 5 days. A probe trial was conducted on day 6, in which the escape platform was removed. For the probe trial, the mouse was placed in the pool at the start location directly opposite of the previous location of the platform during training and allowed to swim for 60 seconds. A one-sample t-test with a theoretical mean of 25% (which is chance) was used to determine significance for the probe trial. A visible platform test was also performed after the probe trial to control for visual acuity. The escape platform was marked with a distinct local cue. A camera was fixed to the ceiling, 1.5 meters from the water surface and connected to a digital tracking device. The tracking information, including time spent in the target (TQ), alternate left (AL), alternate right (AR), and opposite (OP) quadrants, was processed by WaterMaze Software (ActiMetrics, Wilmette, IL). Mice from the control, HF, and HF_16_ groups were used to evaluate the effect of dietary reversal on cognition using MWM. If the mouse did not explore but rather floated upon placement in the water, the data were excluded from the analysis.

### Statistical Analyses

Data analyses were performed using Prism v6 (GraphPad Software, Inc.). All sample sizes represent the number of animals. The differences among groups for the weight, blood glucose, plasma levels, and western immunoblotting data were analyzed using a one-way ANOVA with Tukey’s Multiple Comparison Test. Recognition memory significantly above chance level (50%) was analyzed using a one-sample t-test for the NOR test. Differences between groups (CTRL and HF) in total exploration time were analyzed using a two-tailed t-test. NOR analyses were performed in at least two separate cohorts of mice. For the within subjects NOR, recognition memory was analyzed using a repeated measures two-way ANOVA and Sidak’s multiple comparison test for the 0, 12, and 22 weeks after the start of diet time between groups (CTRL and HF_16_). To determine differences among the percentage of time spent in the quadrants (TQ, AL, OP, and AR) during the MWM probe trial, a one-way ANOVA was performed within each group (CTRL, HF, and HF_16_). All analyses significance was determined using an alpha-level of 0.05.

## Results

### Weight following the dietary reversal of a high-fat diet

**Fig 1:**
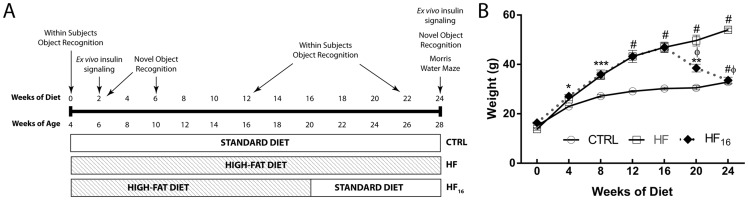
Dietary reversal ameliorates obesity in HF mice. (A) Schematic presentation of the dietary reversal study in mice either on the standard diet or on the high-fat (HF) diet. (B) Body weight after 24 weeks of diet in control (CTRL) mice, HF mice, and mice subjected to dietary reversal after 16 weeks of HF feeding (HF_16_). n = 6 per group. Data represent mean ± standard error of the mean (SEM). *P<0.05 in HF and HF_16_ mice compared with the CTRL mice; **P<0.01 in HF_16_ mice compared with CTRL mice; ***P<0.001 in HF and HF_16_ mice compared with the CTRL mice; #P<0.0001 in HF and HF_16_ mice compared with the CTRL mice; ΦP<0.001 compared with HF mice; #ΦP<0.0001 compared with HF mice.

To evaluate the potential irreversible consequences of a HFD on the hippocampus, we assessed insulin signaling and short- and long-term memory using a unique paradigm of dietary intervention (HF_16_) (Fig 1A). HFD mice (HF and HF_16_) weighed significantly more and consistently gained weight faster than the CTRL mice as early as 4 weeks after the start of the diet (Fig 1B). The HF_16_ mice lost approximately 17.5% of body weight within the first month of dietary intervention (16 to 20 weeks of diet) and 4.6% within the second month (from 20 to 24 weeks of diet; Fig 1B). The weights of the HF_16_ mice after 8 weeks of dietary intervention (24 weeks of diet) were similar to the CTRL mice (Fig 1B).

### Glucose tolerance following the dietary reversal of a high-fat diet

**Fig 2:**
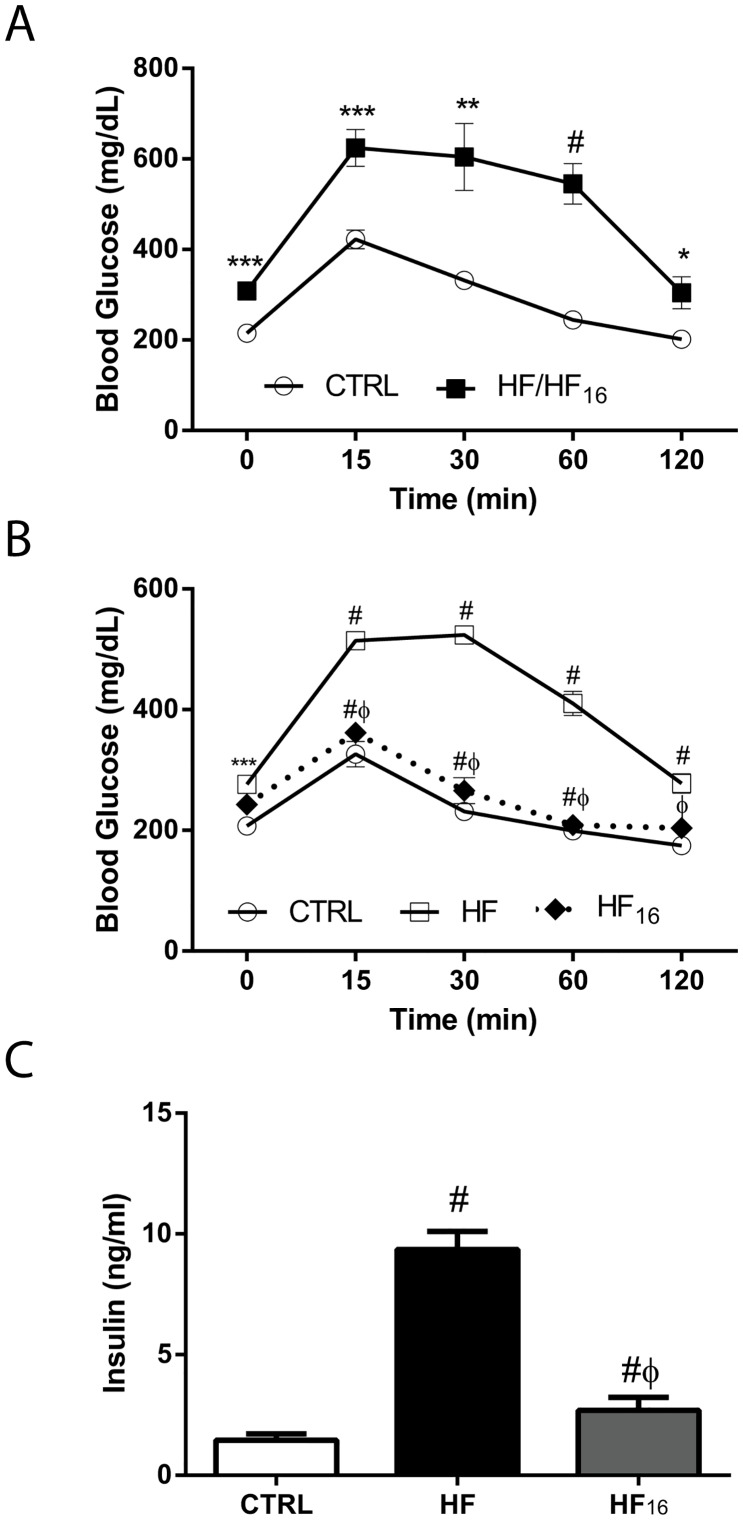
Dietary reversal improves glucose tolerance in HF mice. Impaired glucose tolerance test after (A) 12 weeks of diet in CTRL and HF mice (note: the HF and HF_16_ groups are combined since they are experimentally identical at this time point; n = 6 per group), and (B) 24 weeks of diet in CTRL, HF, and HF_16_ mice (n = 8 per group). (C) Plasma insulin levels in CTRL, HF, and HF_16_ mice after 24 weeks of diet (n = 6 per group). Data represent mean ± SEM. *P<0.05, **P<0.01, ***P<0.001, and #P<0.0001 compared with CTRL mice. ΦP<0.001 and #ΦP<0.0001 compared with HF mice.

To assess insulin sensitivity and levels following dietary reversal, glucose tolerance and plasma insulin levels were assessed. Fasting blood glucose levels were elevated in the HF mice compared with the CTRL mice after 12 and 24 weeks of diet (Fig 2A and 2B). In addition, the blood glucose levels following the administration of a bolus dose of glucose were significantly higher in HF mice compared to CTRL mice after 12 and 24 weeks of diet (Fig 2A and 2B). After eight weeks of dietary intervention, the glucose tolerance levels in the HF_16_ mice were similar to the CTRL mice (Fig 2B). Furthermore, dietary intervention was sufficient to reverse the more than 6-fold increase in the plasma insulin in HF mice after 24 weeks of diet compared with the CTRL mice in the HF_16_ mice (Fig 2C).

### High-fat diet impairs hippocampal insulin signaling

**Fig 3:**
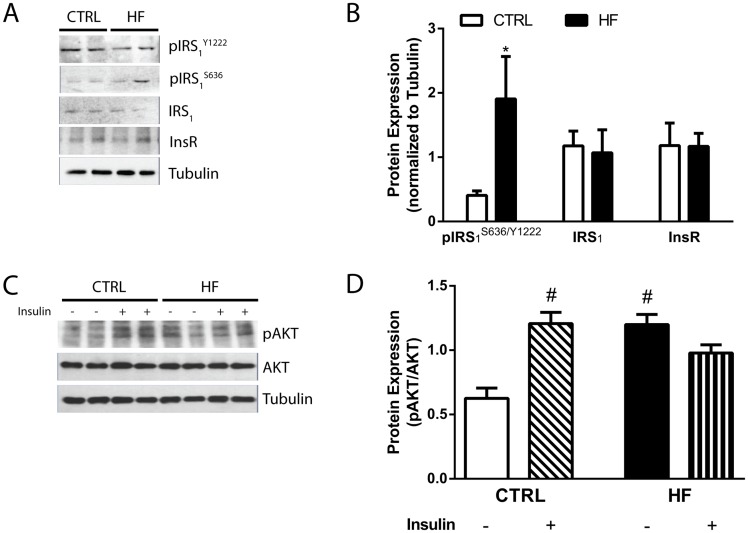
Impaired central insulin signaling after 2 weeks of diet. (A) Representative immunoblots and (B) densitometry of the ratio of the serine phosphorylation of IRS_1_ at the 636 residue (pIRS_1_^S636^) to the tyrosine phosphorylation of IRS_1_ at the 1222 residue (pIRS_1_^Y1222^), IRS_1_, and total insulin receptor (InsR) in CTRL and HF mice after 2 weeks of diet. *P<0.05 compared with CTRL mice. (C) Representative immunoblots and (D) densitometry of pAKT following *ex vivo* insulin stimulation. #P<0.0001 compared with non-insulin treated CTRL mice. All data represent mean ± SEM. n = 6 per group.

The basal protein levels of pIRS_1_, IRS_1_, and InsR were evaluated to assess hippocampal insulin signaling after 2 and 24 weeks of diet. The ability to increase pAKT in response to insulin stimulation was also evaluated at these time points to assess downstream insulin signaling. After 2 weeks of diet, the ratio of basal levels of pIRS_1_^s636^ to pIRS_1_^Y1222^, an index of impaired signaling, were increased nearly four-fold in the HF mice compared with the CTRL mice (Fig 3A and 3B). The basal levels of IRS_1_ and the InsR were not altered after 2 weeks of diet in the HF mice compared with CTRL mice (Fig 3A and 3B). Following insulin stimulation, the protein levels of pAKT increased approximately two-fold in the hippocampus of CTRL mice; however, the levels of pAKT were similar in insulin stimulated and non-stimulated hippocampus of HF mice after 2 weeks of diet (Fig 3C and 3D). Furthermore, the basal levels of pAKT were increased nearly two-fold in the HF mice compared with the CTRL mice after 2 weeks of diet (Fig 3C and 3D).

### Hippocampal insulin signaling following the dietary reversal of a high-fat diet

**Fig 4:**
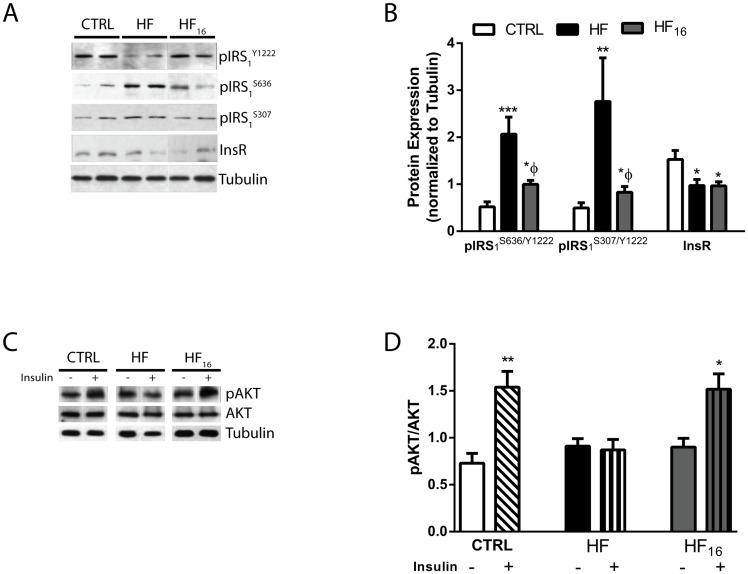
HF diet-induced impairments in central insulin signaling improved with dietary reversal after 24 weeks of diet. (A) Representative immunoblots and (B) densitometry of the ratio of the serine phosphorylation of IRS_1_ at the 636 residue (pIRS_1_^S636^) to the tyrosine phosphorylation of IRS_1_ at the 1222 residue (pIRS_1_^Y1222^), the ratio of the serine phosphorylation of IRS_1_ at the 307 residue (pIRS_1_^S307^) to pIRS_1_^Y1222^, and InsR in CTRL, HF, and HF_16_ mice after 24 weeks of diet. *P<0.05, **P<0.01, and ***P<0.001 compared with CTRL mice; *ΦP<0.05 compared with HF mice. (C) Representative immunoblots and (D) densitometry of pAKT following *ex vivo* insulin stimulation. *P<0.05 and **P<0.01 compared with respective non-insulin treated mice. Data represent mean ± SEM. n = 6 per group.

Similar to 2 weeks after diet, the index of pIRS_1_^S636/Y1222^ and pIRS_1_^S307/Y1222^, representative of impaired insulin signaling, increased nearly three-fold and five-fold, respectively, in the HF mice compared with the CTRL mice after 24 weeks of diet; however, dietary intervention improved this effect in the HF_16_ mice (Fig 4A and 4B). The basal levels of IRS_1_ were not altered after 24 weeks of diet in the HF mice compared with CTRL mice (data not shown). The basal protein levels of the InsR were reduced approximately 1.5-fold in the HF and HF_16_ mice compared with the CTRL mice after 24 weeks of diet (Fig 4A and 4B). Following insulin simulation, the protein levels of pAKT increased approximately two-fold in the hippocampus of CTRL and HF_16_ mice; however, the levels of pAKT were similar in insulin stimulated and non-stimulated hippocampus in HF mice after 24 weeks of diet (Fig 4C and 4D).

### Impact of a high-fat diet on short-term memory

**Fig 5:**
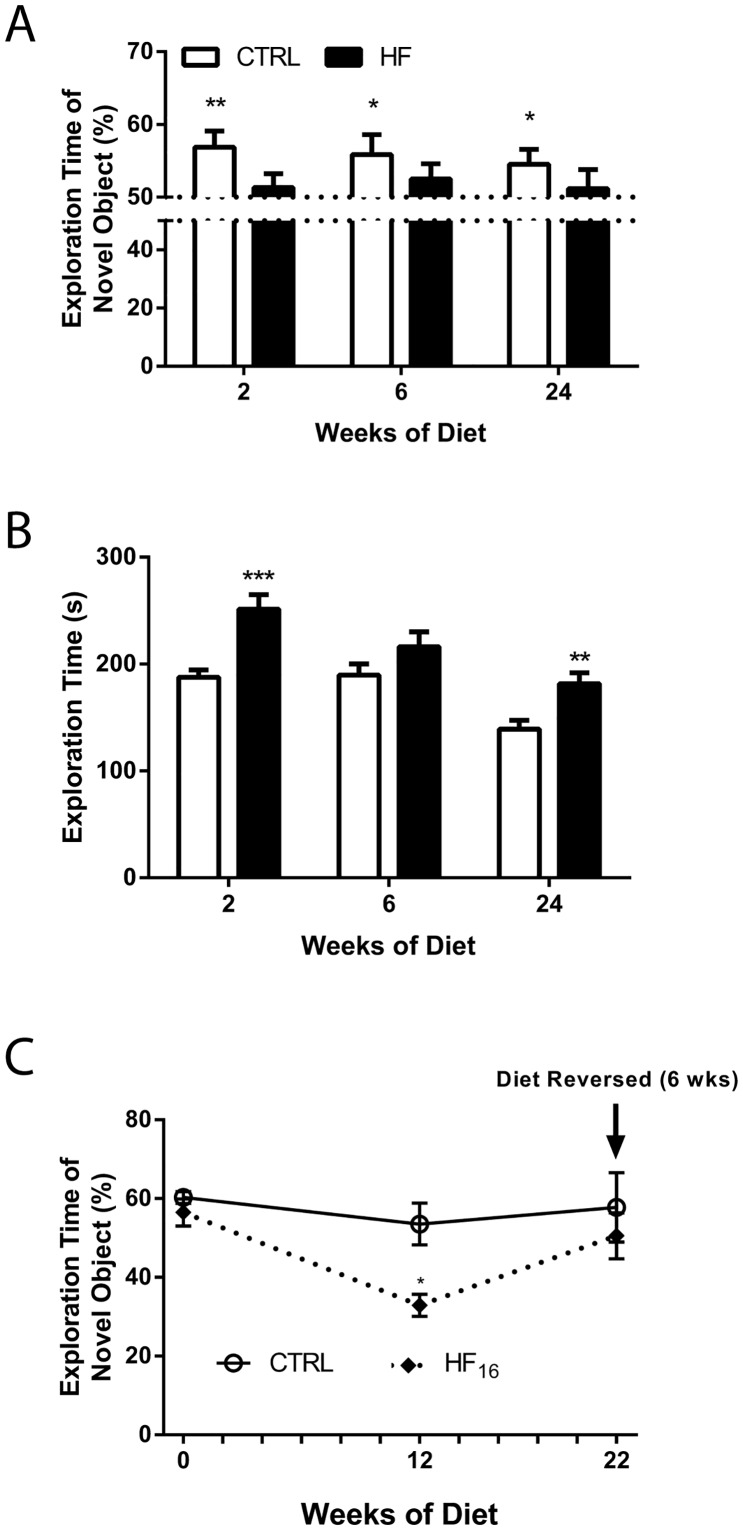
Short-term memory deficits, assessed using novel object recognition test, are reversible. (A) The percentage of time spent exploring the novel object and (B) the total amount of time spent exploring the objects during recognition testing in CTRL and HF mice after 2 (n = 22 and 25, respectively), 6 (n = 22 per group), and 24 (n = 17 and 22, respectively) weeks of diet. *P<0.05, **P<0.01, and ***P<0.001 based on a one-sample t-test. The dotted line represents 50% exploration time. (C) The mean percentage of time spent exploring the novel object for the within subjects object recognition test comparison in CTRL and HF_16_ mice (n = 6 and 9, respectively) prior to diet change (0 weeks) and after 12 and 22 weeks of diet. Data represent mean ± SEM. **P<0.01 compared with mean HF mice at 0 weeks using one-way ANOVA.

To evaluate HFD-induced hippocampal-dependent short-term memory, NOR testing was performed in CTRL and HF mice after 2, 6, and 24 weeks of diet. The CTRL mice spent significantly more than 50% of time exploring the novel object after 2, 6, and 24 weeks of diet; however, the HF mice spent an equal amount of time (50%) exploring the novel and familiar objects at 2, 6, and 24 weeks of diet (Fig 5A). The HF mice spent more total time in exploration than the CTRL mice after 2 and 24 weeks of diet; however, there was no difference in total exploration time after 6 weeks of diet (Fig 5B). To evaluate whether a HFD had a long-lasting impact on recognition memory, a within-subjects study was performed in CTRL and HF_16_ mice. Both the CTRL and HF mice spent approximately 60% of time exploring the novel object prior to the implementation of the HFD (0 weeks) (Fig 5C). The CTRL mice consistently spent approximately 60% of time exploring the novel object after 12 and 22 weeks of diet; however, after 12 weeks of diet the amount of time spent exploring the novel object significantly declined in the HF_16_ mice (Fig 5C). After 6 weeks of dietary intervention on the normal diet, the percentage of time that the HF_16_ mice spent exploring the novel object is comparable to the CTRL mice and the initial (0 weeks) percentage in the mice (Fig 5C).

### Impact of high-fat diet on long-term memory

**Fig 6:**
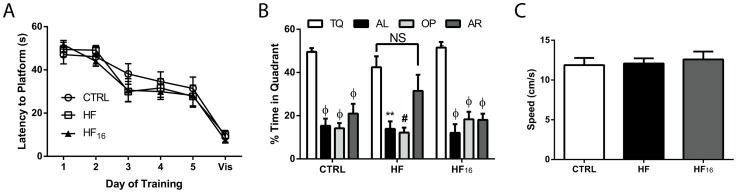
Long-term memory deficits in Morris Water Maze are reversible after prolonged HF feeding. (A) The latency (in seconds) to reach the hidden platform during training and the visible (Vis) platform at the end of testing after 24 weeks of diet in CTRL, HF, and HF_16_ mice (n = 6, 9, and 6 respectively). (B) The percentage of time spent in the target quadrant (TQ) during the probe trial compared to the alternate left (AL; black bars), opposite (OP; light gray bars), and alternate right (AR; dark gray bars) quadrants. (C) The average swimming speed during the probe trial for CTRL, HF, and HF_16_ mice. Data represent mean ± SEM. **P<0.01, #P<0.001; ΦP<0.0001 based on ANOVA.

To evaluate the impact of a HFD on hippocampal-dependent long-term memory, MWM testing was performed after 24 weeks of diet in the CTRL, HF, and HF_16_ mice. The amount of time to find to the hidden platform declined in all groups (CTRL, HF, and HF_16_ mice) over the 5 days of training (Fig 6A). Furthermore, there were no differences among the groups in the latency to reaching the platform when it was clearly marked during the visual platform test (Fig 6A; Vis). During the probe test, the CTRL and HF_16_ mice spent significantly more than 25% of time in the TQ where the platform was previously located; however, the HF mice spent a similar amount of time in the TQ and the AR quadrant searching for the platform (Fig 6B). There is no significant difference in swim speed among the CTRL, HF, and HF_16_ mice (Fig 6C).

## Discussion

According to the Centers for Disease Control and Prevention, sugars and fat contribute to 40% of the daily calories consumed by children and adolescents (ages 2–18). Thus, it is imperative to understand the impact a HFD early in life may have on the hippocampus. In the current study, we investigated the potential irreversible consequences of a HFD early in life on hippocampal insulin signaling and cognitive impairment. To accomplish this, we employed a unique study design of dietary intervention in which mice on HFD were later placed on the standard diet. We show for the first time that dietary intervention improves impaired glucose tolerance, impaired hippocampal insulin signaling, and cognitive deficits; however, the reduction in the levels of the InsRs was not reversible. This suggests that reversing diet, after HFD consumption as a juvenile, may be sufficient to improve both HFD-induced metabolic and cognitive impairment.

It is well established that a HFD leads to weight gain and insulin resistance. Both obesity and peripheral insulin resistance are reversible in man and murine models of obesity after dietary change, exercise, or surgery [33–35]. Thus, the reversal of the obesity phenotype in our HF mice following dietary intervention was expected. This correlated with an improvement of impaired insulin sensitivity, which was evident in the HF mice by hyperinsulinemia and impaired glucose tolerance. Dietary intervention following a HFD early in life normalizes weight, plasma insulin levels, and restores glucose tolerance. Limited data are available demonstrating a correlation between glucose tolerance and hippocampal insulin signaling [36].

A HFD is associated with insulin resistance in various areas of the brain, including the hypothalamus, cortex, and hippocampus [14,37,38]; however, it is not known whether these changes are reversible. Our previous studies demonstrated that HFD induced stress activates c-Jun N-terminal kinase (JNK), which inhibits the tyrosine and activates the serine pIRS_1_ [39–43]. In fact inhibiting JNK, reduces the serine pIRS_1_ at both the 307 and the 636/639 residue [44]. The tyrosine phosphorylation of IRS1 at the 1222 residue is involved in the negative feedback regulation of insulin signaling [45]. Impaired hippocampal insulin signaling in our study is evident by the decrease in the Y1222 pIRS_1_ and the increase in serine 307 and 636/639 pIRS_1_, which has also been reported in animal models of insulin resistance and obesity [46,47]. The increase in the basal levels of pAKT that we observed in the HF mice after 2 weeks of diet is an early indicator of insulin resistance [48]. The decrease in insulin signaling in our study is confirmed by the inability to activate AKT (pAKT) following insulin stimulation, which may lead to a reduction in InsR expression [15]. We did not observe a reduction in InsR expression in the hippocampus after 2 weeks of diet in the HF mice, but rather only after 24 weeks of diet, which suggests that the reduction is likely due to a prolonged reduction in insulin signaling.

Dietary intervention restores tyrosine pIRS_1_ and the ability to activate AKT following insulin stimulation; however, the decrease in InsR expression is not reversed with dietary intervention. This is the first study, to our knowledge, that reports an improvement in impaired hippocampal insulin signaling and an irreversible decrease InsR expression in the hippocampus with dietary intervention. It is possible that the reduction in InsR expression in the hippocampus following a HFD is a protective mechanism to prevent over activation of insulin signaling and subsequent desensitization. The levels of InsRs are reduced in the brains of patients with Alzheimer’s disease, in aged rodents, and in rodents with diabetes [49–51]. Hence, it is possible that a HFD leads to an acceleration of age-related decrease in InsR expression. Although further studies are warranted, this may be a factor in the increased risk of age-related neurodegenerative diseases in MetS [52].

A role for insulin in cognition has been implicated, specifically in spatial memory [20,53,54]. The HF mice in the current study display deficits in both NOR and MWM. This is consistent with previous studies with the implementation of a HFD early in life in rodents [24–26,55–57]. These previous studies reported recognition memory deficits at 20 weeks after the start of a HFD. Deficits in recognition memory were observed using another task, the novel object location, at 8 weeks after the start of a HFD [58]. Furthermore, these deficits in recognition memory remained despite 5 additional weeks of 70% calorically restricted HFD. In the current study, we observed memory impairment at multiple time points and as early as 2 weeks after the start of the HFD. The impairments in memory improved within 6 weeks of dietary intervention. To our knowledge, this is the first report demonstrating that HFD-induced deficits in both short-and long-term memory improve with dietary intervention. Future studies will address whether age-related cognitive deficits appear earlier in mice that have been exposed to a HFD, despite dietary intervention.

It is likely that multiple mechanisms contribute to HFD-induced cognitive impairment. Hippocampal-dependent cognitive deficits induced by HFD have been attributed to leptin, calcium dysregulation, inflammation, cellular stress, and impaired insulin signaling [57–64]. Our previous studies have implicated the involvement of cellular stress in impaired hippocampal insulin signaling [65–67]. Previous studies have implicated a role of the tumor necrosis factor alpha (TNFα) in both the activation of cellular stress and impaired insulin signaling [68–71]. Although we did not observe a difference in TNFα in HF mice after 24 weeks of diet (S1 Fig), it is possible that TNFα mediates these studies suggest that obesity results in a feed forward cycle that includes endoplasmic reticulum stress, inflammation, and impaired insulin signaling, which may impact cognition. Enhancing insulin signaling has promising therapeutic benefits for improving cognition [22,72]; however, more research is needed to understand its impact on endoplasmic reticulum stress and inflammation. We demonstrate that impaired glucose tolerance and hippocampal insulin signaling correlate with memory deficits. We show for the first time that switching mice from a HF to standard diet improves several factors associated with the MetS, hippocampal insulin signaling alterations, and cognitive deficits.

It is not known which metabolic factor(s) contribute to cognitive impairment; however, our previous study demonstrated that endoplasmic reticulum stress and impaired insulin signaling were only present in models of MetS with hyperglycemia/impaired glucose tolerance [65]. We demonstrate that HFD-induced impairments in cognition, glucose tolerance and hippocampal insulin signaling are improved with a drastic change in dietary fat intake. On the other hand, despite a drastic change in diet, the InsR expression levels in the hippocampus remain reduced following a HFD. The long-term impact of this decrease in InsR expression has yet to be determined. The results of this study are likely clinically relevant to a large population of children and adolescents who are exposed to HFDs early in life despite making healthier choices as adults.

## Supporting Information

S1 Fig The potential impact of inflammation on impaired insulin signaling. Densitometry analysis of tumor necrosis factor alpha (TNFα) after 24 weeks of diet in mice on a standard (STD) and high-fat (HFD) diet. n = 5 per group.
(DOCX)

S2 Fig The potential involvement of the Ras/ERK pathway in memory impairment. Representative immunoblot of phosphorylated extracellular signal-regulated kinases (pERK) after 24 weeks of diet in mice on a standard (control; CTRL) and high-fat (HFD) diet.
(DOCX)

S1 Table Caloric content of the standard and high-fat diets. The distribution of protein, carbohydrates, and fat content in the standard (control; CTRL) and high-fat (HF) diet. Food Intake was measured for one week after 18 weeks of diet (22 weeks of age) in CTRL, HF, and the dietary reversal group, HF_16_ (16 weeks of a HF diet and 2 weeks of dietary reversal). This data was collected from a separate cohort of animals that are not used in any of the analyses presented.
(DOCX)

## Abbreviations

AL: alternate left
AR: alternate right
CTRL: control
HF: high-fat
HFD: high-fat diet
InsR: insulin receptor
IRS_1_: insulin receptor substrate 1
MetS: metabolic syndrome
MWM: Morris Water Maze
NOR: novel object recognition
OP: opposite
PI3K: phosphatidylinositol 3-kinase
p: phosphorylation
AKT: protein kinase B
TQ: target quadrant
Vis: visible
