# Supplementary material for: Dietary Reversal Ameliorates Short- and Long-Term Memory Deficits Induced by High-fat Diet Early in Life
Source: PLoS One. 2016 Sep 27;11(9):e0163883. doi: 10.1371/journal.pone.0163883 (PMC5038939; doi:10.1371/journal.pone.0163883)
Supplement: S1 Fig — Densitometry analysis of tumor necrosis factor alpha (TNFα) after 24 weeks of diet in mice on a standard (STD) and high-fat (HFD) diet. n = 5 per group. (DOCX) [file pone.0163883.s001.docx]

**S1 Figure. The potential impact of inflammation on impaired insulin signaling.** Densitometry analysis of tumor necrosis factor alpha (TNFα) after 24 weeks of diet in mice on a standard (STD) and high-fat (HFD) diet. n=5 per group.
