# Supplementary material for: Dietary Reversal Ameliorates Short- and Long-Term Memory Deficits Induced by High-fat Diet Early in Life
Source: PLoS One. 2016 Sep 27;11(9):e0163883. doi: 10.1371/journal.pone.0163883 (PMC5038939; doi:10.1371/journal.pone.0163883)
Supplement: S2 Fig — Representative immunoblot of phosphorylated extracellular signal-regulated kinases (pERK) after 24 weeks of diet in mice on a standard (control; CTRL) and high-fat (HFD) diet. (DOCX) [file pone.0163883.s002.docx]

**S2 Figure. The potential involvement of the Ras/ERK pathway in memory impairment.** Representative immunoblot of phosphorylated extracellular signal-regulated kinases (pERK) after 24 weeks of diet in mice on a standard (control; CTRL) and high-fat (HFD) diet.

**
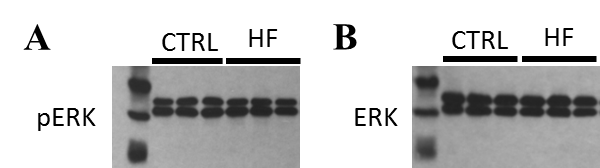
**
