# Supplementary material for: Dietary Reversal Ameliorates Short- and Long-Term Memory Deficits Induced by High-fat Diet Early in Life
Source: PLoS One. 2016 Sep 27;11(9):e0163883. doi: 10.1371/journal.pone.0163883 (PMC5038939; doi:10.1371/journal.pone.0163883)
Supplement: S1 Table — The distribution of protein, carbohydrates, and fat content in the standard (control; CTRL) and high-fat (HF) diet. Food Intake was measured for one week after 18 weeks of diet (22 weeks of age) in CTRL, HF, and the dietary reversal group, HF16 (16 weeks of a HF diet and 2 weeks of dietary reversal). This data was collected from a separate cohort of animals that are not used in any of the analyses presented. (DOCX) [file pone.0163883.s003.docx]

**S1 Table. Caloric content of the standard and high-fat diets**

|  | **CTRL** | | **HF** | | **HF_16_** |
| --- | --- | --- | --- | --- | --- |
|  | **D12450B** | | **D05090701** | |  |
|  | **gram** | **kcal** | **gram** | **kcal** |  |
| **Protein** | 19 | 20 | 25 | 20 | - |
| **Carbohydrate** | 67 | 70 | 32 | 26 | - |
| **Fat** | 4 | 10 | 30 | 54 | - |
| **kcal/gm** | **3.85** | - | **5.04** | - | - |
| **Food Intake (kcal/day)** | **42.2** | | **61.9** | | **38.3** |

The distribution of protein, carbohydrates, and fat content in the standard (control; CTRL) and high-fat (HF) diet. Food Intake was measured for one week after 18 weeks of diet (22 weeks of age) in CTRL, HF, and the dietary reversal group, HF_16_ (16 weeks of a HF diet and 2 weeks of dietary reversal). This data was collected from a separate cohort of animals that are not used in any of the analyses presented.
